# Supplementary material for: High-content fluorescence imaging with the metabolic flux assay reveals insights into mitochondrial properties and functions
Source: Commun Biol. 2020 May 29;3:271. doi: 10.1038/s42003-020-0988-z (PMC7260371; doi:10.1038/s42003-020-0988-z)
Supplement: Supplementary file 1 — Supplementary Information [file 42003_2020_988_MOESM1_ESM.pdf]

SUPPLEMENTARY MATERIAL

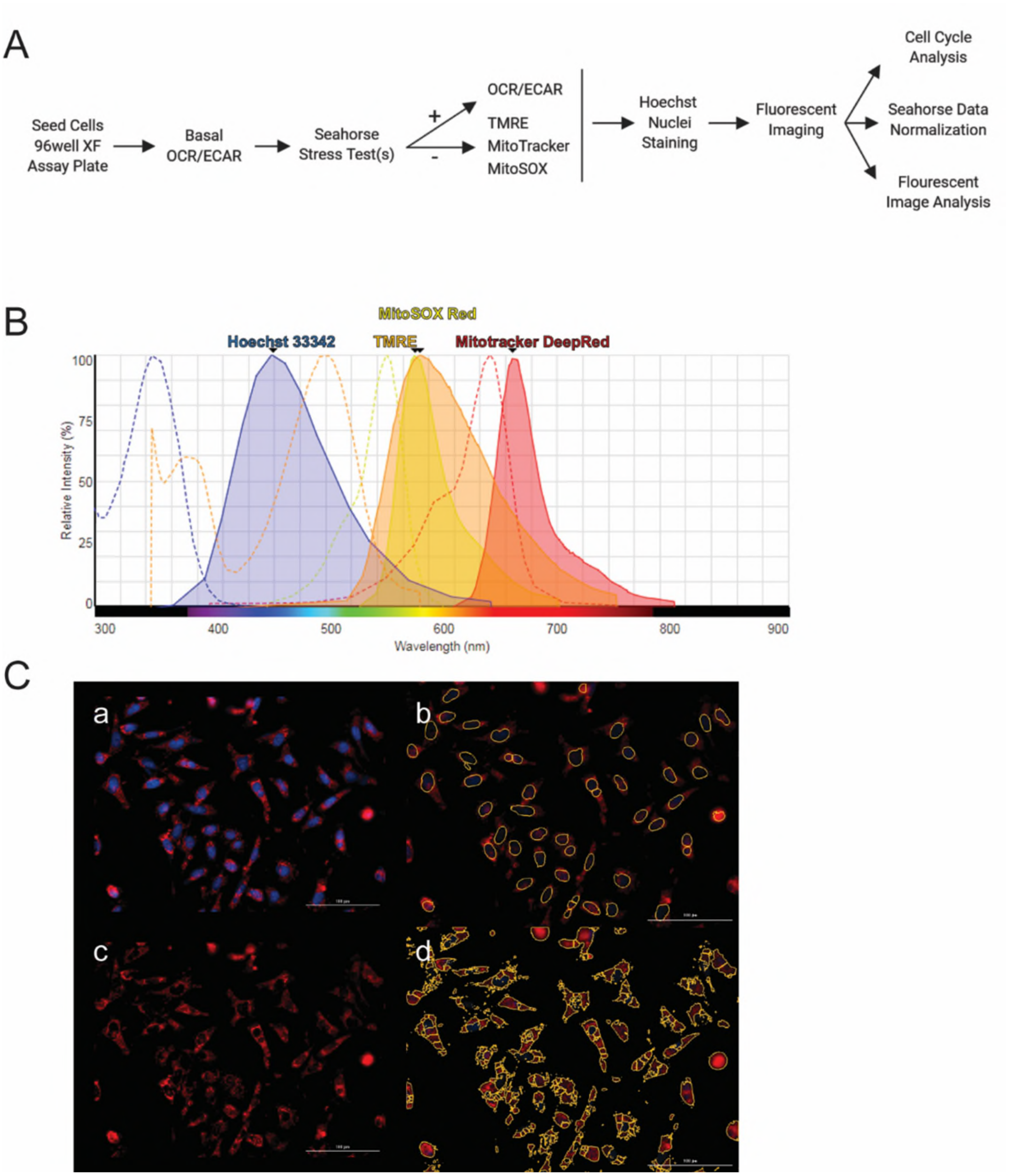

Supplemental Fig. 1 ***Seahorse-Cytation assay workflow and imaging acquisition and analysis parameters.*** (**A**) Seahorse-*Cytation 5* workflow schematic*.* (**B**) Emission spectra of the fluorescent stains utilized in this study. (**C**) Hoechst nuclei staining and nuclei masking (a,b) and Mitotracker Red fluorescent staining and its applied secondary mask for image analysis and quantification (c,d).

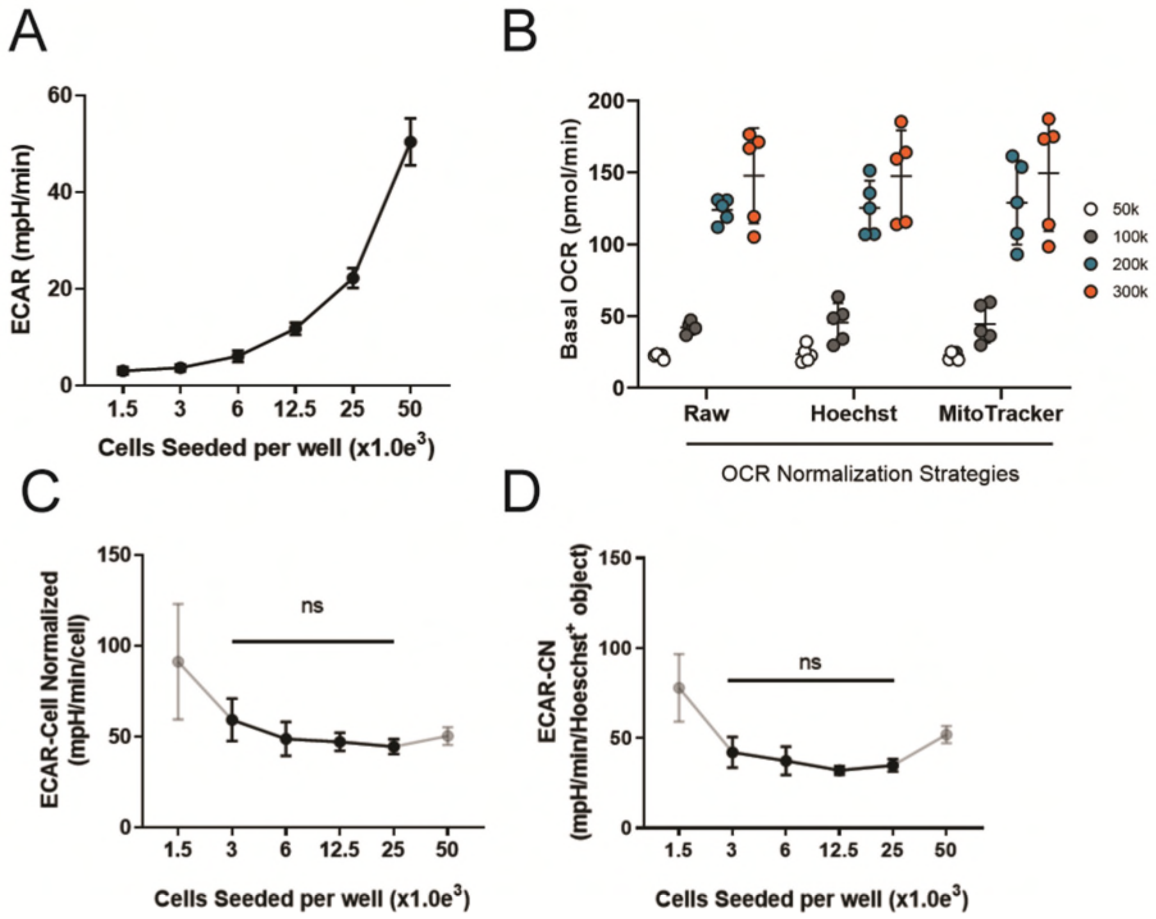

Supplemental Fig. 2 ***ECAR results based on differing normalization strategies.*** (**A**) Raw ECAR values increase with cell density. (**B**) $CD4^+$ T-cell basal OCR data normalization strategies (i.e. raw – total cell input; Hoechst – total fluorescently labeled nuclei; MitoTracker – normalization based on mitochondrial units per cell) based on increasing seeded cell densities i.e. 50,000 = 50k, etc. Correcting for cell density with either traditional hematocytometer (**C**) or fluorescent-based nuclei imaging (**D**) has little impact on ECAR data normalization post Mito Stress Test. n.s., non-significant

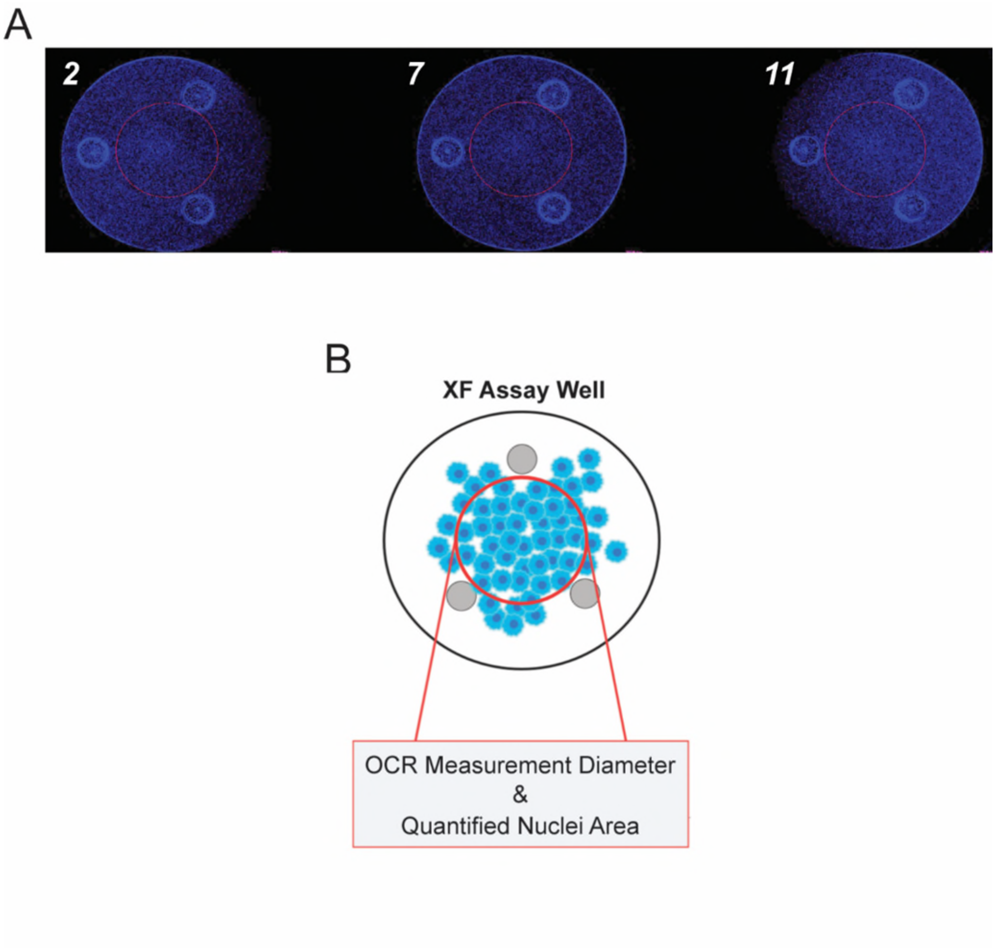

Supplemental Fig. 3 ***XF assay edge effects and imaging parameters***. (**A**) Representative images of fluorescent nuclei imaging (blue) revealing edge effects in the XF 96 well cell culture plate. Red circle implicates where bioanalyzer data is traditionally captured. Numbers indicate column on XF 96 well plate. (**B**) Schematic representation of a XF 96 cell culture well where respiration measurements and fluorescent nuclei images are obtained. Cells are in blue; sensors are in gray.

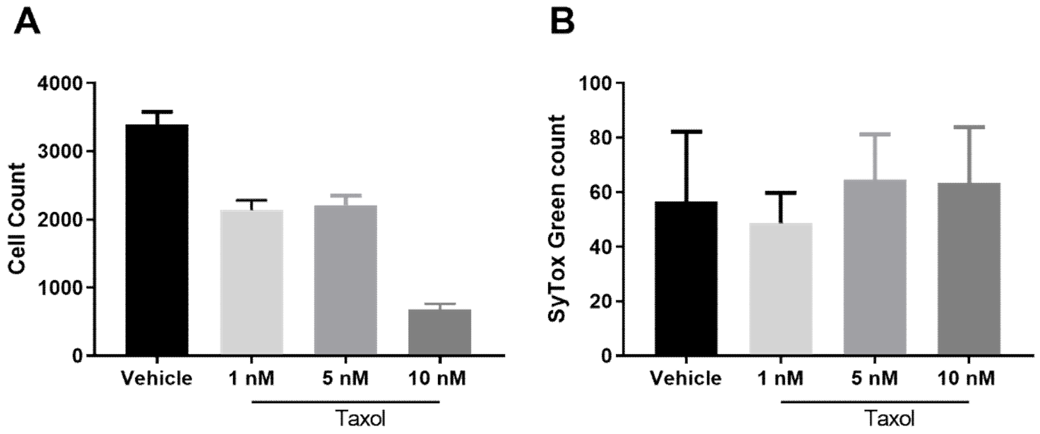

Supplemental Fig. 4 ***Low dose taxol treatment arrests cell cycle but does not induce significant toxicity***. (**A**) Cell number in taxol treated samples. (**B**) Cell viability by SyTox green staining in taxol treated samples.

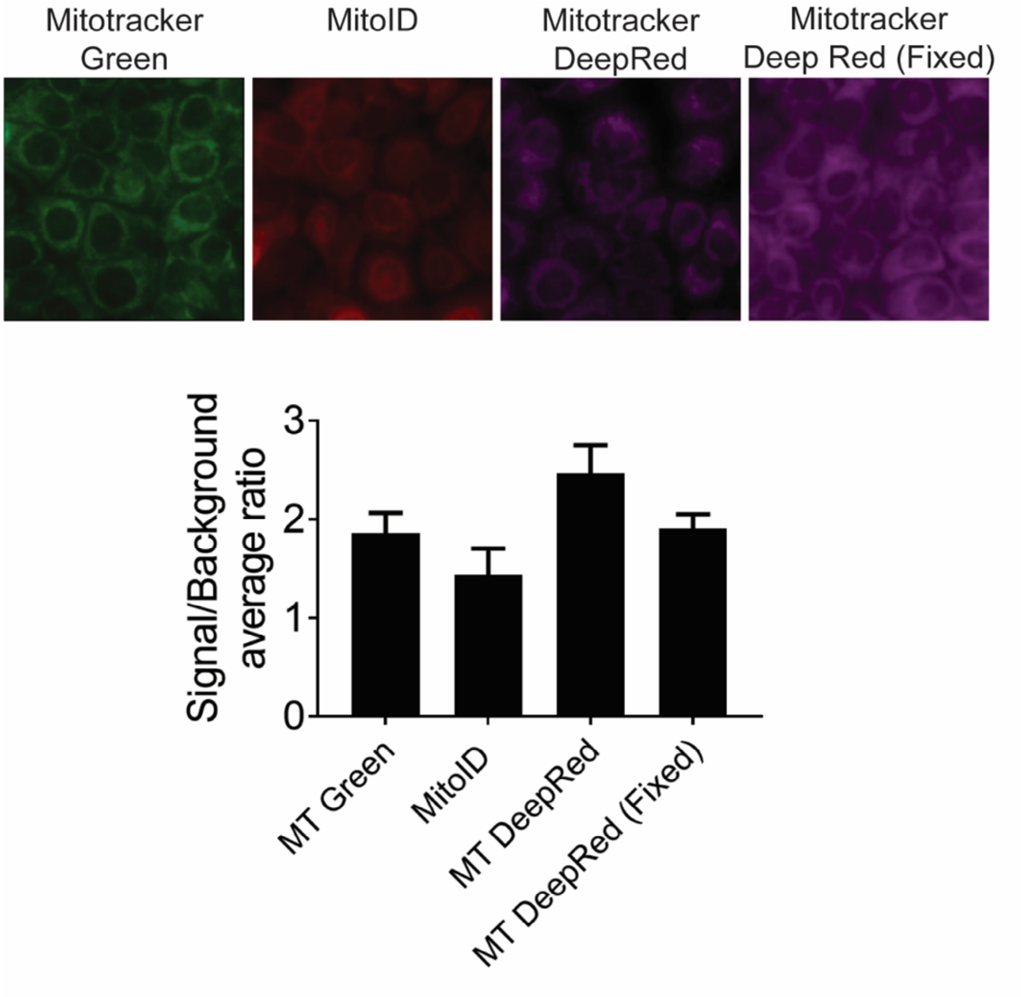

Supplemental Fig. 5 ***Evaluation of various Mitotracker dyes and determination of their signal to noise background.*** Mitotracker Green, MitoID, and Mitotracker DeepRed were evaluated in living cells while Mitotracker DeepRed was also evaluated post fixation (i.e. “Fixed”). Representative fluorescent images at top. The quantified signal to background ratio is presented at bottom.

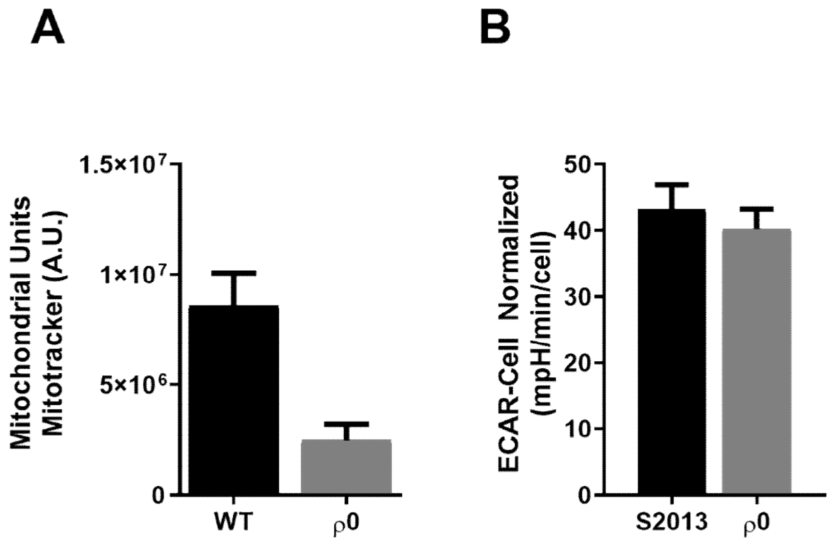

Supplemental Fig. 6 ***Rho0 S2-013 cells (S2-013-ρ0) mitochondrial quantitation and ECAR values***. (**A**) S2-013 wild type (WT) pancreatic cancer cells harbor greater numbers of mitochondria than their S2-013-ρ0 (Rho0) counterparts, as quantified by total Mitotracker fluorescence intensity. Data displayed as arbitrary fluorescent units (A.U.). (**B**) ECAR measurements in S2-013-ρ0 vs WT cells. Data corrected for overall cell numbers.

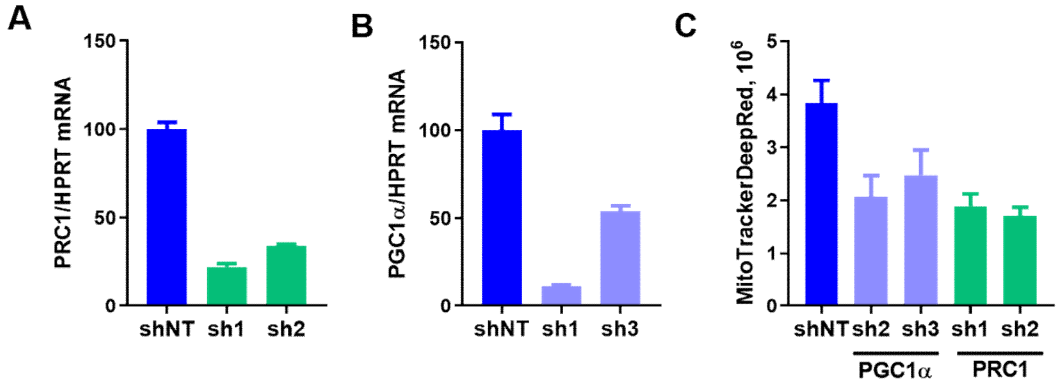

Supplemental Fig. 7 ***PRC1 and PGC-1a RNAi knockdown characterization.*** Confirmation of PRC1 (**A**) and PGC-1a (**B**) knockdown by short hairpin (sh)RNA as measured by qPCR in T3M4 shPRC1 and shPGC-1a cells. NT, non-targeting. (**C**) Total levels of mitochondria are lower in PGC1a and PRC1 knockdown cells as determined by Mitotracker fluorescent staining.

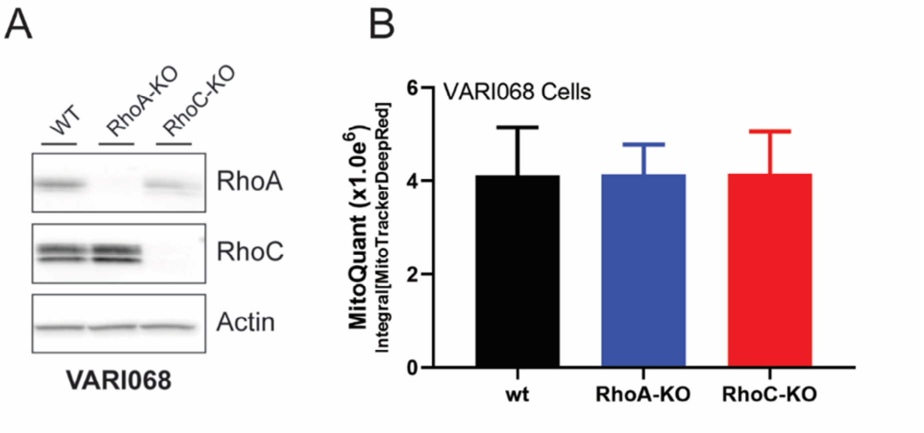

Supplemental Fig. 8 ***Protein expression and mitochondrial quantification in the Rho knockout VARI068 breast cancer cell model.*** (**A**) Protein expression of RhoA or RhoC in CRISPR knockout breast cancer PDX cell line, VARI068. (**B**) Mitochondrial quantitation as determined by Mitotracker fluorescent staining and imaging in wild type, RhoA KO, and RhoC KO VARI068 cells.

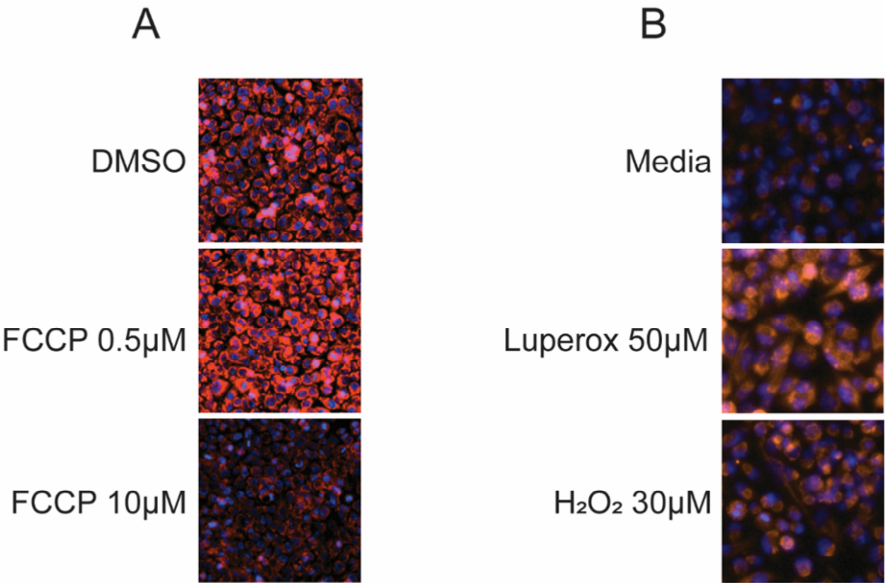

Supplemental Fig. 9 ***TMRE and MitoSOX fluorescent images.*** Representative fluorescent images utilized for image analysis of (**A**) TMRE post FCCP treatment (**B**) or MitoSOX imaging post Luperox/H_2O_2 stimulation.

Supplementary Table 1. **shRNA constructs utilized for this study.**

Denomination	Gene Name	TRCN#
**PGC1α-sh1**	PPARGC1a	TRCN0000364084
**PGC1α-sh2**	PPARGC1a	TRCN0000364087
**PGC1α-sh3**	PPARGC1a	TRCN0000364086
**PRC1-sh1**	PPRC1	TRCN0000063296
**PRC1-sh2**	PPRC1	TRCN0000437395
**NT**	Non-Targeting	CCG GCA ACA AGA TGA AGA GCA CCA ACT CGA GTT GGT GCT CTT CAT CTT GTT GTT TTT

Supplementary Table 2. **RT-qPCR primer sequences utilized for this study.**

**HPRT-Fw**	TGACACTGGCAAAACAATGCA
**HPRT-Rv**	GGTCCTTTTCACCAGCAAGCT
**PARGC1A-Fw**	ACCATATTCCAGGTCAAGATCAA
**PPARGC1A-Rv**	GGCTTGACTCATAGTAATAGCAGGA
**PPRC1-Fw**	TGGCATCACTTGAGGATGAG
**PPRC1-Rv**	AGCCCTCAGGCAGTGTCA

***Supplementary Table 3*. Statistical data summary for comparisons made in Figure 2E.**

	Mean Diff.	95.00% CI of diff.	Significance	Summary	Adjusted P Value
G1					
Vehicle vs. 1 nM	16.000	12.014 to 19.986	Yes	****	0.000000000000015
Vehicle vs. 5 nM	15.000	11.014 to 18.986	Yes	****	0.000000000000021
Vehicle vs. 10 nM	41.000	37.014 to 44.986	Yes	****	<0.000000000000001
1 nM vs. 5 nM	-1.0000	-4.9861 to 2.9861	No	ns	0.913702907587112
1 nM vs. 10 nM	25.000	21.014 to 28.986	Yes	****	<0.000000000000001
5 nM vs. 10 nM	26.000	22.014 to 29.986	Yes	****	<0.000000000000001
S					
Vehicle vs. 1 nM	-3.0000	-6.9861 to 0.98605	No	ns	0.208140545392875
Vehicle vs. 5 nM	-1.0000	-4.9861 to 2.9861	No	ns	0.913702907587112
Vehicle vs. 10 nM	-2.0000	-5.9861 to 1.9861	No	ns	0.559051072923722
1 nM vs. 5 nM	2.0000	-1.9861 to 5.9861	No	ns	0.559051072923722
1 nM vs. 10 nM	1.0000	-2.9861 to 4.9861	No	ns	0.913702907587112
5 nM vs. 10 nM	-1.0000	-4.9861 to 2.9861	No	ns	0.913702907587112
G2/M					
Vehicle vs. 1 nM	-11.000	-14.986 to -7.0139	Yes	****	0.000000000503183
Vehicle vs. 5 nM	-12.000	-15.986 to -8.0139	Yes	****	0.000000000018863
Vehicle vs. 10 nM	-33.000	-36.986 to -29.014	Yes	****	<0.000000000000001
1 nM vs. 5 nM	-1.0000	-4.9861 to 2.9861	No	ns	0.913702907587112
1 nM vs. 10 nM	-22.000	-25.986 to -18.014	Yes	****	<0.000000000000001
5 nM vs. 10 nM	-21.000	-24.986 to -17.014	Yes	****	<0.000000000000001

Supplementary Table 4. **Statistical data summary for comparisons made in Figure 3F.**

Granulated	Mean Diff.	95.00% CI of diff.	Significance Summary		Adjusted P Value
DMSO vs. 1.8	0.00	-4.45 to 4.45	No	ns	>0.999999999999999
DMSO vs. 8	10.8	6.35 to 15.3	Yes	****	0.000023669331293
DMSO vs. 40	9.80	5.35 to 14.3	Yes	****	0.000088802348254
1.8 vs. 8	10.8	6.35 to 15.3	Yes	****	0.000023669331293
1.8 vs. 40	9.80	5.35 to 14.3	Yes	****	0.000088802348254
8 vs. 40	-1.00	-5.45 to 3.45	No	ns	0.650481546911509
Fragmented					
DMSO vs. 1.8	-3.00	-7.45 to 1.45	No	ns	0.179554138090012
DMSO vs. 8	-12.2	-16.7 to -7.75	Yes	****	0.000003672026368
DMSO vs. 40	-12.0	-16.5 to -7.55	Yes	****	0.000004792101784
1.8 vs. 8	-9.20	-13.7 to -4.75	Yes	***	0.000194572245507
1.8 vs. 40	-9.00	-13.5 to -4.55	Yes	***	0.000252180019586
8 vs. 40	0.200	-4.25 to 4.65	No	ns	0.927683149467141
