# Supplementary material for: High-content fluorescence imaging with the metabolic flux assay reveals insights into mitochondrial properties and functions
Source: Commun Biol. 2020 May 29;3:271. doi: 10.1038/s42003-020-0988-z (PMC7260371; doi:10.1038/s42003-020-0988-z)
Supplement: Supplementary file 3 — Reporting Summary [file 42003_2020_988_MOESM3_ESM.pdf]

nature research

Corresponding author(s): Costas A. Lyssiotis

Last updated by author(s): Feb 21, 2020

Reporting Summary

Nature Research wishes to improve the reproducibility of the work that we publish. This form provides structure for consistency and transparency in reporting. For further information on Nature Research policies, see Authors & Referees and the Editorial Policy Checklist.

Statistics

For all statistical analyses, confirm that the following items are present in the figure legend, table legend, main text, or Methods section.

n/a	Confirmed	
☐	☒	The exact sample size (*n*) for each experimental group/condition, given as a discrete number and unit of measurement
☐	☒	A statement on whether measurements were taken from distinct samples or whether the same sample was measured repeatedly
☐	☒	The statistical test(s) used AND whether they are one- or two-sided<br>*Only common tests should be described solely by name; describe more complex techniques in the Methods section.*
☒	☐	A description of all covariates tested
☐	☒	A description of any assumptions or corrections, such as tests of normality and adjustment for multiple comparisons
☐	☒	A full description of the statistical parameters including central tendency (e.g. means) or other basic estimates (e.g. regression coefficient) AND variation (e.g. standard deviation) or associated estimates of uncertainty (e.g. confidence intervals)
☐	☒	For null hypothesis testing, the test statistic (e.g. *F*, *t*, *r*) with confidence intervals, effect sizes, degrees of freedom and *P* value noted<br>*Give P values as exact values whenever suitable.*
☒	☐	For Bayesian analysis, information on the choice of priors and Markov chain Monte Carlo settings
☒	☐	For hierarchical and complex designs, identification of the appropriate level for tests and full reporting of outcomes
☒	☐	Estimates of effect sizes (e.g. Cohen's *d*, Pearson's *r*), indicating how they were calculated

Our web collection on statistics for biologists contains articles on many of the points above.

Software and code

Policy information about availability of computer code

Data collection	No custom code was used to collect data for this study.
Data analysis	Seahorse data was analyzed post hoc in Microsoft excel and Graphpad Prism. Imaging data analysis was performed with algorithms built into the Biotek Gen5 software. Instructions for how to properly apply these algorithms are described in detail in the Gen5 software manual.

For manuscripts utilizing custom algorithms or software that are central to the research but not yet described in published literature, software must be made available to editors/reviewers. We strongly encourage code deposition in a community repository (e.g. GitHub). See the Nature Research guidelines for submitting code & software for further information.

Data

Policy information about availability of data

All manuscripts must include a data availability statement. This statement should provide the following information, where applicable:

- Accession codes, unique identifiers, or web links for publicly available datasets
- A list of figures that have associated raw data
- A description of any restrictions on data availability

The data presented herein are not publicly available since these studies are largely proof of principle concepts, although, the primary data is available from the C.A.L. upon request.

Field-specific reporting

Please select the one below that is the best fit for your research. If you are not sure, read the appropriate sections before making your selection.

☒ Life sciences ☐ Behavioural & social sciences ☐ Ecological, evolutionary & environmental sciences

For a reference copy of the document with all sections, see nature.com/documents/nr-reporting-summary-flat.pdf

Life sciences study design

All studies must disclose on these points even when the disclosure is negative.

Sample size	Sample sizes, on a per-experimental basis, where chosen to be as large as possible within the confines of the experimental design e.g. utilizing 96 wells in a 96 well plate. Each experiment sought to obtain the greatest amount of information possible, therefore we fully maximized our technical replicates when applicable.
Data exclusions	n/a
Replication	each experiment was performed two or more times, independently to ensure reproducibility. Experiments that did not replicate where not included in these results.
Randomization	Each experimental design started with the same amount of cells, seeded at the same time under the same conditions. We then randomly selected areas of the 96-well plate to perform various staining conditions e.g. mitosox, mitotracker, etc.
Blinding	Experiments were blinded in various manners throughout the study.

Reporting for specific materials, systems and methods

We require information from authors about some types of materials, experimental systems and methods used in many studies. Here, indicate whether each material, system or method listed is relevant to your study. If you are not sure if a list item applies to your research, read the appropriate section before selecting a response.

Materials & experimental systems

n/a	Involved in the study	
☒	☐	Antibodies
☐	☒	Eukaryotic cell lines
☒	☐	Palaeontology
☒	☐	Animals and other organisms
☒	☐	Human research participants
☒	☐	Clinical data

Methods

n/a	Involved in the study	
☒	☐	ChIP-seq
☒	☐	Flow cytometry
☒	☐	MRI-based neuroimaging

Eukaryotic cell lines

Policy information about cell lines

Cell line source(s)	MIA PaCa-2 were obtained from ATCC. Pa-Tu-8902 cells were obtained from DSMZ. S2-103 and T3M4 cells were generously provided by Anguraj Sadanandam (ICR, UK). VARI068 cells were generated from a patient derived xenograft in the Merajver laboratory.
Authentication	STR profiled
Mycoplasma contamination	tested bimonthly for mycoplasma contamination
Commonly misidentified lines (See ICLAC register)	n/a
